# Comparing the effects of different dynamic sitting strategies in wheelchair seating on lumbar-pelvic angle

**DOI:** 10.1186/s12891-016-1358-3

**Published:** 2016-12-09

**Authors:** Chun-Ting Li, Yao-Te Peng, Yen-Ting Tseng, Yen-Nien Chen, Kuen-Horng Tsai

**Affiliations:** 1 Graduate Institute of Mechatronic System Engineering, National University of Tainan, No. 33, Sec. 2, Shu-Lin St., West Central Dist., Tainan City 70005 Taiwan; 2 Department of BioMedical Engineering, National Cheng Kung University, No.1, University Rd., East Dist., Tainan City, 70101 Taiwan; 3 Center of Excellence for Diagnostic Products, Biomedical Technology and Device Research Laboratories, Industrial Technology Research Institute, No. 195, Sec. 4, Chung-Hsing Rd., Chutung Township, Hsinchu County 31040 Taiwan

**Keywords:** Lower back pain, Lumbar spine, Wheelchair, Dynamic sitting

## Abstract

**Background:**

Prolonged static sitting in a wheelchair is associated with an increased risk of lower back pain. The wheelchair seating system is a key factor of this risk because it affects spinal loading in the sitting position. In this study, 7 dynamic sitting strategies (DSSs) are examined: lumbar prominent dynamic sitting (LPDS), back reclined dynamic sitting (BRDS), femur upward dynamic sitting (FUDS), lumbar prominent with back reclined dynamic sitting (LBDS), lumbar prominent with femur upward dynamic sitting (LFDS), back reclined with femur upward dynamic sitting (BFDS), and lumbar prominent with back reclined with femur upward dynamic sitting (LBFDS). The objective of this study was to analyze the biomechanical effects of these sitting strategies on lumbar-pelvic angles.

**Methods:**

Twenty able-bodied participants were recruited for the study. All participants performed LPDS, BRDS, FUDS, LBDS, LFDS, BFDS, and LBFDS in a random order. All lumbar-pelvic angle parameters, including the static lumbar angle, static pelvic angle, lumbar range of motion, and pelvic range of motion were measured and compared.

**Results:**

Results show that LBDS and LBFDS enabled the most beneficial lumbar movements, although the difference between the 2 strategies was nonsignificant. BRDS and BFDS enabled the most beneficial pelvic movements, although the difference between the 2 strategies was nonsignificant. Among all the upright DSSs, LPDS and LFDS enabled the most beneficial lumbar and pelvic movements, although no significant difference was observed between these 2 strategies.

**Conclusions:**

We identified the effects and differences among 7 DSSs on lumbar-pelvic angles. Wheelchair users can choose the most suitable DSS that meets their needs. These findings may serve as a reference for practicing physicians or wheelchair users to choose an appropriate dynamic wheelchair seating system.

**Trial registration:**

ISRCTN12389808, 18th November 2016, retrospectively registered.

## Background

One of the causes of mechanical lower back pain is prolonged and abnormal stress exerted on tissues surrounding the lumbar, pelvis, and/or femur [1–3]. The resulting creep effect stimulates surrounding nociceptors and causes discomfort or pain [1, 4–6]. The loading from prolonged static sitting is associated with an increased risk of lower back pain [1, 3, 7]. In particular, people with lower limb disorders who rely on prolonged wheelchair use for mobility are at a high risk of lower back pain [3, 8, 9].

Previous studies have found that wheelchair users often sit in a position that causes lumbar kyphosis with posterior pelvic tilt [3, 10]. Prolonged lumbar kyphosis causes creep in the spinal ligaments and fascia; as little as 5 min can cause an approximately 40% decrease in the ability of the intervertebral ligaments to protect the intervertebral discs [1, 6, 11]. Some wheelchair users use lumbar support to help maintain normal lumbar lordosis [3, 10, 12]. However, lumbar lordosis transfers stress to the posterior annulus fibrosus, anterior longitudinal ligament, facet joints, and spinous process [1, 13–15]. However, this phenomenon negatively affects stress concentration on the posterior annulus fibrosus, unless the disc is severely degenerated and narrowed [1, 16–18]. Both lumbar kyphosis and lordosis produce creep load on surrounding soft tissues, decreasing the ability of the intervertebral discs to distribute stress evenly, reducing the distance between the vertebral arches, and increasing the risk of disc degeneration and herniation [1, 6, 14, 19]. Previous studies have shown that avoiding prolonged lumbar kyphosis and lordosis can help prevent lower back pain [1, 20, 21]. In addition, movements that produce lumbar kyphosis and lordosis cause different rates of metabolite transport in the anterior annulus fibrosus, nucleus pulposus, and posterior annulus fibrosus [1, 22]. The U.S. Department of Health suggests shifting body weight, such as by lumbar extension or flexion movements, every 15 min to prevent tissue damage [21, 23]. However, this is difficult for wheelchair users who are incapable of autonomous lumbar movement.

Previous studies have proposed numerous dynamic devices for relieving lumbar loading, such as dynamic lumbar supports, dynamic reclined backrests, and dynamic ischial/femur cushions [20, 21, 24–27]. Findings have confirmed that such devices can periodically adjust the sitting position, stimulate body movement, and improve the loading from prolonged static sitting [20, 21, 24–27]. In clinical observations, many wheelchair users employ more than one of these pressure-relieving devices simultaneously because their functions do not conflict with one another. To date, no study has examined whether combining these pressure-relieving devices produces a positive or negative effect.

**Fig. 1:**
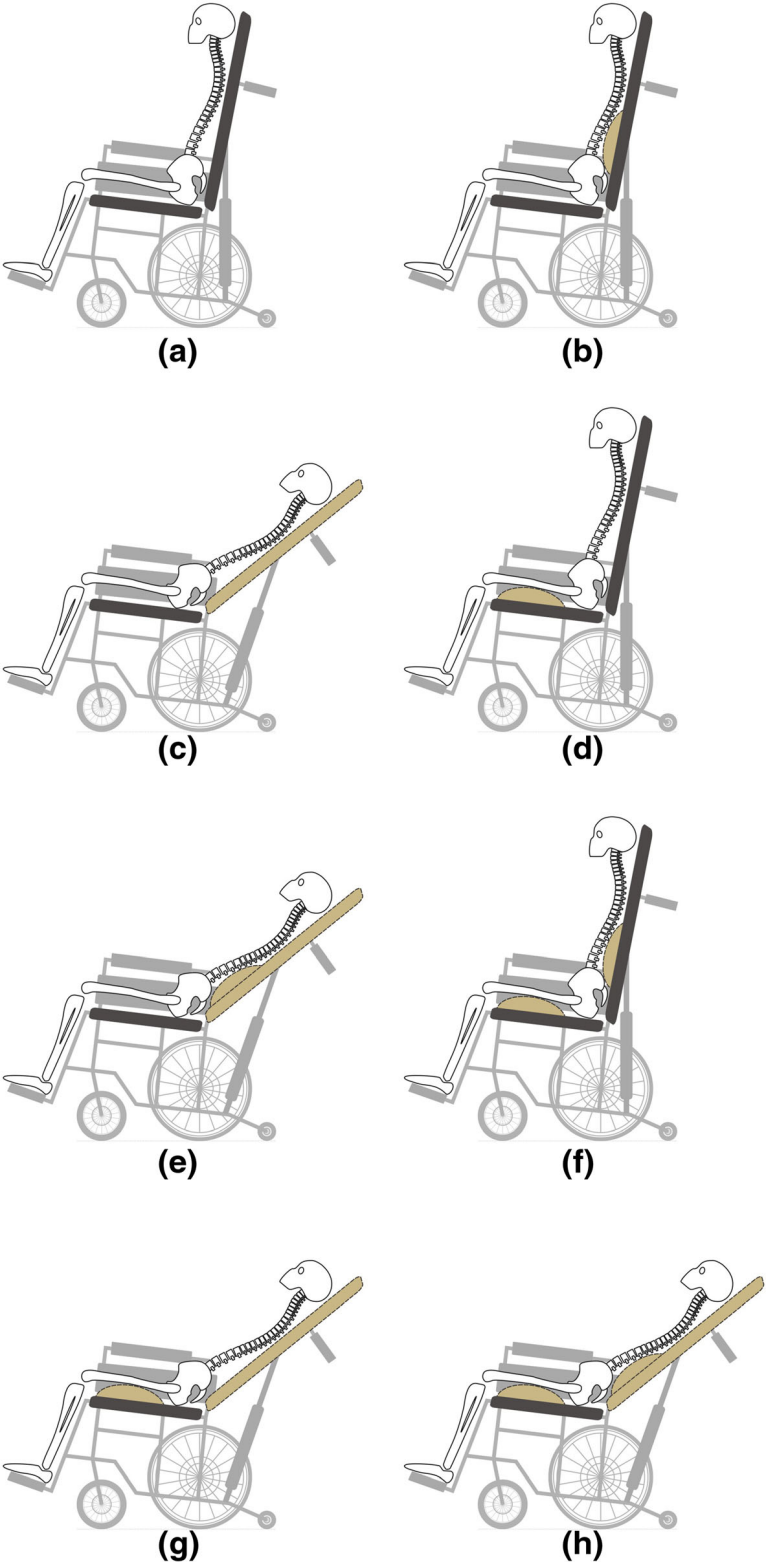
Seven different dynamic sitting strategies. **a** to **b** dynamic change was lumbar prominent dynamic sitting (LPDS), **a** to **c** dynamic change was back reclined dynamic sitting (*BRDS*), **a** to **d** dynamic change was femur upward dynamic sitting (*FUDS*), **a** to **e** dynamic change was lumbar prominent with back reclined dynamic sitting (*LBDS*), **a** to **f** dynamic change was lumbar prominent with femur upward dynamic sitting (*LFDS*), **a** to **g** dynamic change was back reclined with femur upward dynamic sitting (*BFDS*), and **a** to **h** dynamic change was lumbar prominent with back reclined with femur upward dynamic sitting (*LBFDS*)

Regarding the preceding description, we tested three typical dynamic pressure-relieving devices individually and compared them in four clinically common combinations, yielding a total of seven dynamic sitting strategies (DSSs): lumbar prominent dynamic sitting (LPDS), back reclined dynamic sitting (BRDS), femur upward dynamic sitting (FUDS), lumbar prominent with back reclined dynamic sitting (LBDS), lumbar prominent with femur upward dynamic sitting (LFDS), back reclined with femur upward dynamic sitting (BFDS), and lumbar prominent with back reclined with femur upward dynamic sitting (LBFDS), as shown in Fig. 1. We quantified their effects on the lumbar-pelvic angle and examined whether they can effectively promote periodic lumbar movement and help lower the risk of lower back pain.

## Methods

### Participants

Twenty able-bodied people were recruited to participate in this study (11 men, 9 women; age, 22.3 ± 1.7 years old; weight, 62.0 ± 11.4 kg; height, 168.1 ± 9.1 cm; body mass index, 21.8 ± 2.9 kg/m^2^). The participants were able-bodied people with no known spinal pathology or musculoskeletal disorder and had not sought medical treatment for lumbar pain within the previous 6 months. They were asked to refrain from all types of resistance exercise within 48 h before the experimental start. All participants read and signed an informed consent form that explained the research objectives and experimental protocol. This study was approved by the Institutional Review Board of National Cheng Kung University Hospital.

### Wheelchair

An experimental wheelchair was developed for this study. The wheelchair was equipped with a lumbar adjustment module, femur adjustment module, and backrest tilt mechanism. The lumbar adjustment module and femur adjustment module each contained a programmable air bag. Customized microprocessors were used to control the magnitude and frequency of air bag inflation and deflation. Each air bag was 40 × 23 cm^2^ and 4-cm thick when fully inflated. The backrest tilt mechanism was fitted with a programmable screw rod. The tilt angle and frequency of the backrest were controlled through a customized microprocessor. The backrest can be tilted from 90° ~ 160°. In addition, the position of the lumber adjustment module, position of the femur adjustment module, depth of the seat cushion, length of the footrests, and angle of the footrests can be adjusted according to each participant’s body type and dimensions. Furthermore, a 1-cm-thick foam pad was installed on the backrest and seat cushion to minimize skin contact with uneven surfaces in the backrest and seat cushion, which might cause discomfort to some participants.

### Strategies

This study proposes 7 DSSs, as shown in Fig. 1. The experimental wheelchair settings for each DSS are detailed as follows: (1) LPDS: The lumbar adjustment module is positioned at L3 of participant, and the air bag provides dynamic adjustment by deflating to 0 cm and inflating to 4 cm at periodic intervals. (2) BRDS: Upper body contact is maintained with the backrest in the experimental wheelchair, and the backrest tilt mechanism provides dynamic adjustment by tilting backward and forward between 100° and 150° at periodic intervals. (3) FUDS: The femur adjustment module is positioned at the midpoint of the participant’s femur, and the air bag provides dynamic adjustment by deflating to 0 cm and inflating to 4 cm at periodic intervals. (4) LBDS: This combines the LPDS and BRDS settings. (5) LFDS: This combines the LPDS and FUDS settings. (6) BFDS: This combines the BRDS and FUDS settings. (7) LBFDS: This combines the LPDS, BRDS, and FUDS settings.

### Protocol

The initial settings for the experimental wheelchair formed a 100° angle between the backrest and seat cushion, and a 120° angle between the seat cushion and footrest. The seat cushion was adjusted to allow a gap between the cushion and popliteal fossa. When the participants were seated in the experimental wheelchair, they were asked to rest their upper body against the backrest, relax their arms and place them at their sides, try to keep their thighs parallel to the ground, place their feet firmly on top of the footrest at shoulder width, and look directly ahead [10, 28]. Next, they performed each of the 7 DSSs in random order. Each DSS test lasted 20 min, with periodic changes at 5-min intervals. The participants were asked to stand up and move around for 5 min between each DSS test.

### Measurement

**Fig. 2:**
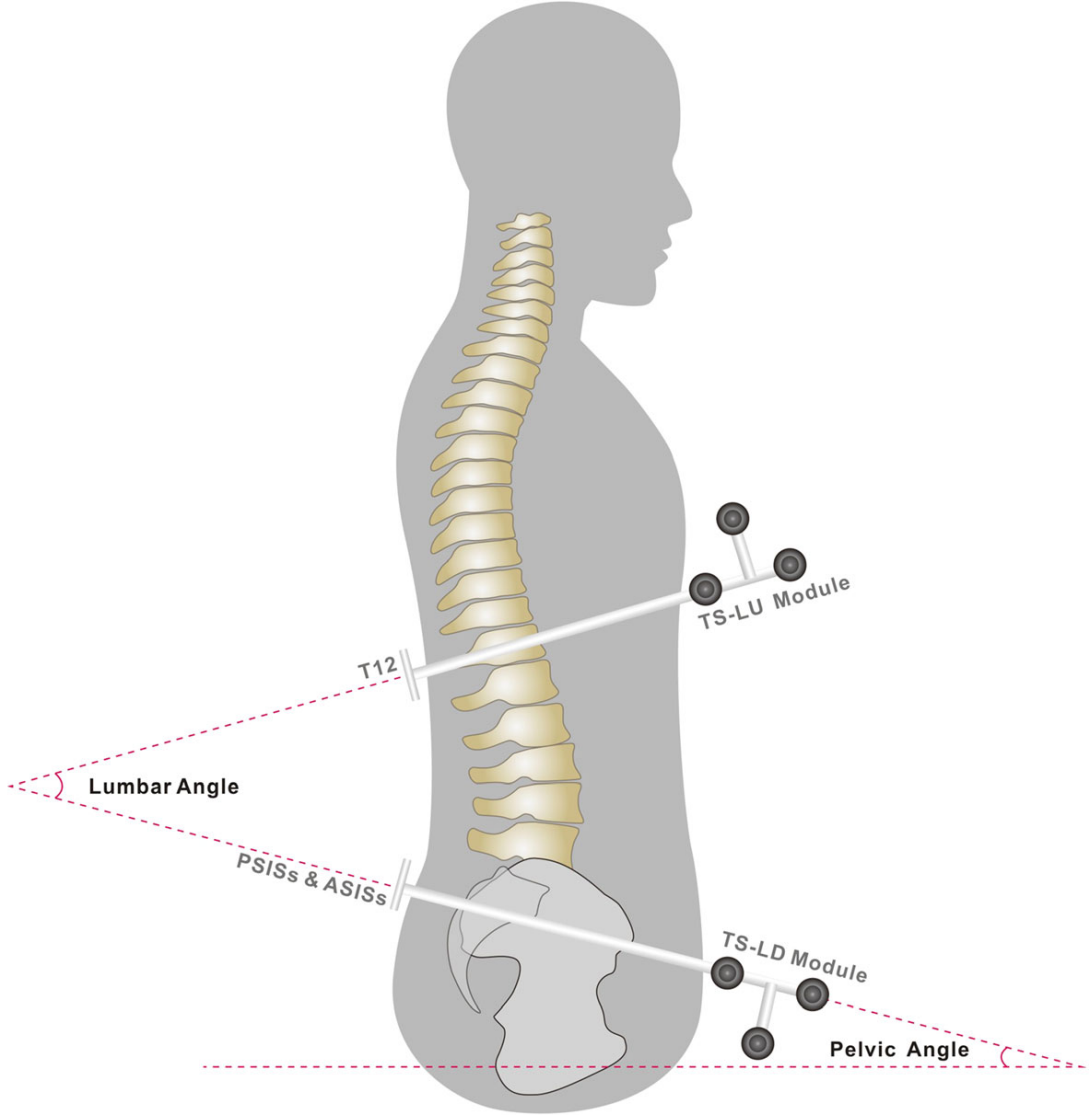
Lumbar-pelvic angle illustration. The miniature ultrasound transmission modules, TS-LU module was placed firmly around the T12 level, TS-LD module was situated around the level of the posterior superior iliac spines and the anterior superior iliac spines (PSISs & ASISs). Lumbar angle was obtained from the angle between the TS-LU module and the TS-LD module; pelvic angle was obtained by measuring the angle between the TS-LD module and the horizontal plane

**Fig. 3:**
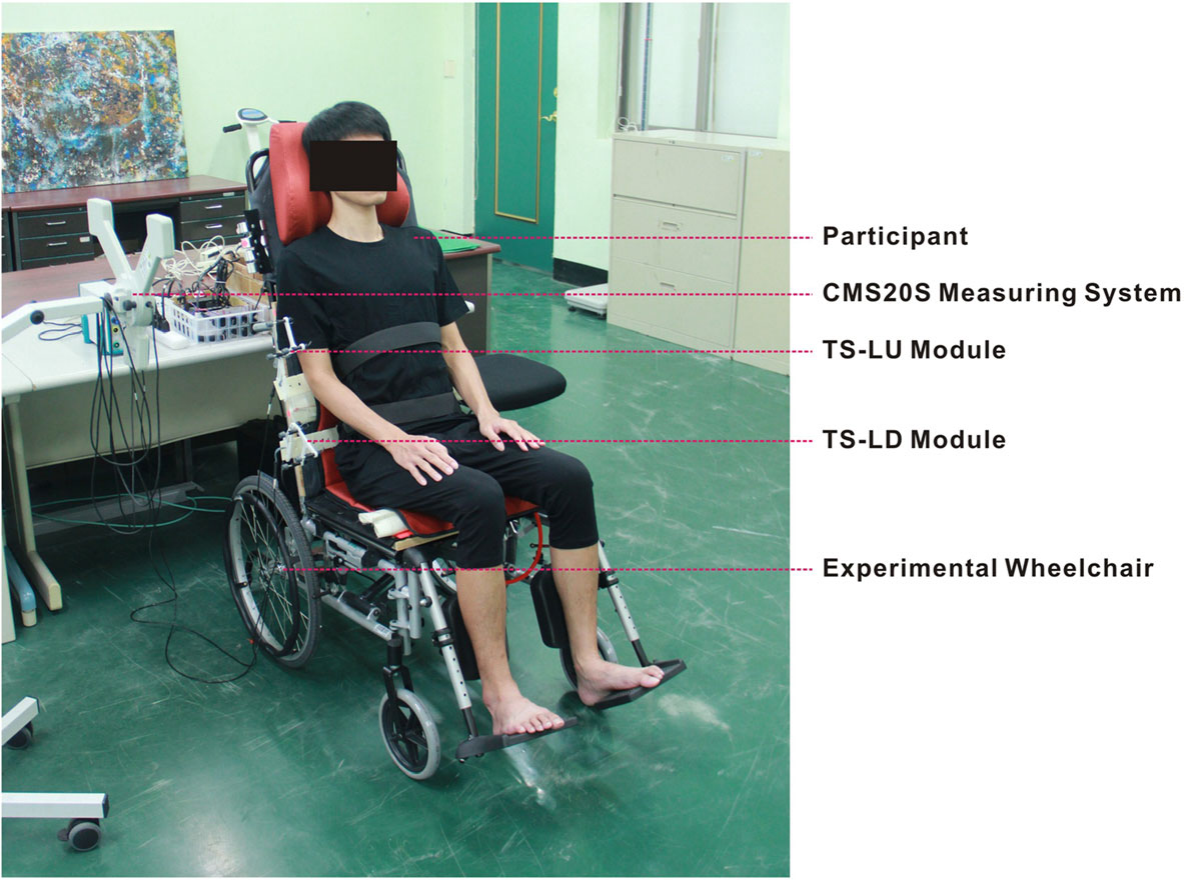
Experimental setup. The picture shows the experimental setup with participant, experimental wheelchair, CMS20S Measuring System, miniature ultrasound transmission modules

An ultrasound-based motion analysis system (CMS20S Measuring System; zebris Medical GmbH, Isny im Allgäu, Germany) was used to measure the participants’ lumbar-pelvic angles including the static lumbar angle (LA) and static pelvic angle (PA) after dynamic changes, and the lumbar range of motion (LRM) and pelvic range of motion (PRM) resulting from dynamic changes. Previous studies have shown that the CMS20S Measuring System has high reliability [29, 30]. It comprises one ultrasound signal receiver and 2 miniature ultrasound transmission modules. The transmission modules (attachment set with triple markers TS-LU and TS-LD; zebris Medical GmbH, Isny im Allgäu, Germany) were attached at T12 and at the pelvis (the posterior superior iliac spines and the anterior superior iliac spines), as shown in Fig. 2 and Fig. 3. Lumbar-pelvic angle parameters were calculated using WinData software (WinData, version 2.22.25; zebris Medical GmbH, Isny im Allgäu, Germany). The sampling frequency was set to 30 Hz. Prior to the experiments involving measurements of lumbar-pelvic angles, all ultrasound sensors were arranged in a row on a vertical mounting bracket and the sensors were zero corrected. LA and LRM data were derived from the angle between the TS-LU and TS-LD modules; PA and PRM data were derived from the TS-LD module angle relative to a horizontal plane, as shown in Fig. 2. All parameters (LA, PA, LRM, and PRM) used degree as the unit of measurement.

### Statistics

SPSS Version 17 (SPSS Institute, Chicago, IL, USA) was used for all statistical analyses. All parameters, LA, PA, LRM, and PRM were compared among the 7 DSSs (LPDS, BRDS, FUDS LBDS, LFDS, BFDS, and LBFDS) through a Friedman test. A Wilcoxon signed-rank test was used to detect statistically significant differences in the dependent variables across the tests. The level of statistical significance was set at *P* < 0.05.

## Results

All the participants completed the lumbar-pelvic angle measurements according to the LPDS, BRDS, FUDS, LBDS, LFDS, BFDS, and LBFDS strategies. No participant reported adverse reactions to the experimental protocol.

The results of LA are shown in Table 1. Compared with the LPDS strategy, the BRDS, FUDS, and BFDS appeared to yield significantly lower LA (*P* < 0.001), the LBDS and LBFDS appeared to yield significantly higher LA (*P* ≤ 0.002), and no significant difference with LFDS was observed. Compared with the BRDS strategy, the FUDS appeared to yield significantly lower LA (*P* = 0.001), the LBDS, LFDS, and LBFDS appeared to yield significantly higher LA (*P* < 0.001), and no significant difference with BFDS was observed. Compared with the FUDS strategy, the LBDS, LFDS, BFDS, and LBFDS appeared to yield significantly higher LA (*P* < 0.001). Compared with the LBDS strategy, the LFDS and BFDS appeared to yield significantly lower LA (*P* ≤ 0.001), and no significant difference with LBFDS was observed. Compared with the LFDS strategy, the BFDS appeared to yield significantly lower LA (*P* = 0.001) and the LBFDS appeared to yield significantly higher LA (*P* < 0.001). When compared with the BFDS strategy, the LBFDS appeared to yield significantly higher LA (*P* < 0.001).

**Table 1:** Static lumbar angle after dynamic change

		*P* value of the Wilcoxon signed-rank test					
DSSs	LA (Degree)	LPDS	BRDS	FUDS	LBDS	LFDS	BFDS
LPDS	16.14 ± 5.98						
BRDS	6.13 ± 4.75	<0.001					
FUDS	−1.94 ± 4.91	<0.001	0.001				
LBDS	26.99 ± 9.68	0.002	<0.001	<0.001			
LFDS	16.43 ± 6.16	0.550	<0.001	<0.001	0.001		
BFDS	6.56 ± 2.99	<0.001	0.794	<0.001	<0.001	0.001	
LBFDS	27.26 ± 8.36	0.001	<0.001	<0.001	0.654	<0.001	<0.001
*P* value of the Friedman test		<0.001					

Comparison of mean static lumbar angle (*LA*) after dynamic change across 7 dynamic sitting strategies (*DSSs*), which include Lumbar Prominent Dynamic Sitting (*LPDS*), Back Reclined Dynamic Sitting (*BRDS*), Femur Upward Dynamic Sitting (*FUDS*), Lumbar Prominent with Back Reclined Dynamic Sitting (*LBDS*), Lumbar Prominent with Femur Upward Dynamic Sitting (*LFDS*), Back Reclined with Femur Upward Dynamic Sitting (*BFDS*), and Lumbar Prominent with Back Reclined with Femur Upward Dynamic Sitting (*LBFDS*). Values are mean ± standard deviation (*N* = 20). The positive value (+) represents the lumbar lordosis while the negative value (−) represents the lumbar kyphosis

The results of PA are shown in Table 2. Compared with the LPDS strategy, the BRDS, FUDS, LBDS, BFDS, and LBFDS appeared to yield significantly lower PA (*P* < 0.001), and no significant difference with LFDS was observed. Compared with the BRDS strategy, the FUDS, LBDS, LFDS, and LBFDS appeared to yield significantly higher PA (*P* ≤ 0.001), and no significant difference with BFDS was observed. Compared with the FUDS strategy, the LBDS, BFDS, and LBFDS appeared to yield significantly lower PA (*P* < 0.001) and the LFDS appeared to yield significantly higher PA (*P* < 0.001). Compared with the LBDS strategy, the LFDS appeared to yield significantly higher PA (*P* < 0.001), the BFDS appeared to yield significantly lower PA (*P* = 0.011), and no significant difference with LBFDS was observed. Compared with the LFDS strategy, the BFDS and LBFDS appeared to yield significantly lower LA (*P* < 0.001). When compared with the BFDS strategy, the LBFDS appeared to yield significantly higher PA (*P* = 0.008).

**Table 2:** Static pelvic angle after dynamic change

		*P* value of the Wilcoxon signed-rank test					
DSSs	PA (Degree)	LPDS	BRDS	FUDS	LBDS	LFDS	BFDS
LPDS	3.60 ± 3.82						
BRDS	−51.78 ± 4.07	<0.001					
FUDS	−11.17 ± 3.56	<0.001	<0.001				
LBDS	−43.35 ± 7.97	<0.001	<0.001	<0.001			
LFDS	3.11 ± 3.36	0.191	<0.001	<0.001	<0.001		
BFDS	−50.10 ± 8.95	<0.001	0.823	<0.001	0.011	<0.001	
LBFDS	−44.16 ± 7.94	<0.001	0.001	<0.001	0.654	<0.001	0.008
*P* value of the Friedman test		<0.001					

Comparison of mean static pelvic angle (*PA*) after dynamic change across 7 dynamic sitting strategies (*DSSs*), which include Lumbar Prominent Dynamic Sitting (*LPDS*), Back Reclined Dynamic Sitting (*BRDS*), Femur Upward Dynamic Sitting (*FUDS*), Lumbar Prominent with Back Reclined Dynamic Sitting (*LBDS*), Lumbar Prominent with Femur Upward Dynamic Sitting (*LFDS*), Back Reclined with Femur Upward Dynamic Sitting (*BFDS*), and Lumbar Prominent with Back Reclined with Femur Upward Dynamic Sitting (*LBFDS*). Values are mean ± standard deviation (*N* = 20). The positive value (+) represents the pelvic anterior tilt while the negative value (−) represents the pelvic posterior tilt

The results of LRM are shown in Table 3. Compared with the LPDS strategy, the BRDS, FUDS, and BFDS appeared to yield significantly lower LRM (*P* ≤ 0.033), the LBDS and LBFDS appeared to yield significantly higher LRM (*P* = 0.001), and no significant difference with LFDS was observed. Compared with the BRDS strategy, the FUDS appeared to yield significantly lower LRM (*P* < 0.001), the LBDS, LFDS, and LBFDS appeared to yield significantly higher LRM (*P* ≤ 0.008), and no significant difference with BFDS was observed. Compared with the FUDS strategy, the LBDS, LFDS, BFDS, and LBFDS appeared to yield significantly higher LRM (*P* < 0.001). Compared with the LBDS strategy, the LFDS and BFDS appeared to yield significantly lower LRM (*P* ≤ 0.001), and no significant difference with LBFDS was observed. Compared with the LFDS strategy, the BFDS appeared to yield significantly lower LRM (*P* = 0.004) and the LBFDS appeared to yield significantly higher LRM (*P* = 0.001). When compared with the BFDS strategy, the LBFDS appeared to yield significantly higher LRM (*P* < 0.001).

**Table 3:** Range of motion in lumbar angle form dynamic change

		*P* value of the Wilcoxon signed-rank test					
DSSs	LRM (Degree)	LPDS	BRDS	FUDS	LBDS	LFDS	BFDS
LPDS	13.28 ± 6.74						
BRDS	8.82 ± 4.24	0.033					
FUDS	1.49 ± 2.47	<0.001	<0.001				
LBDS	29.48 ± 8.83	0.001	<0.001	<0.001			
LFDS	13.54 ± 6.82	0.940	0.008	<0.001	0.001		
BFDS	8.52 ± 3.72	0.005	0.478	<0.001	<0.001	0.004	
LBFDS	28.78 ± 8.46	0.001	<0.001	<0.001	0.305	0.001	<0.001
*P* value of the Friedman test				<0.001			

Comparison of mean lumbar range of motion (*LRM*) form dynamic change across 7 dynamic sitting strategies (*DSSs*), which include Lumbar Prominent Dynamic Sitting (*LPDS*), Back Reclined Dynamic Sitting (*BRDS*), Femur Upward Dynamic Sitting (*FUDS*), Lumbar Prominent with Back Reclined Dynamic Sitting (*LBDS*), Lumbar Prominent with Femur Upward Dynamic Sitting (*LFDS*), Back Reclined with Femur Upward Dynamic Sitting (*BFDS*), and Lumbar Prominent with Back Reclined with Femur Upward Dynamic Sitting (*LBFDS*). Each LRM parameter is given as the averaging value when two dynamic alteration process over a sitting trial. Values are mean ± standard deviation (*N* = 20)

The results of PRM are shown in Table 4. Compared with the LPDS strategy, the BRDS, LBDS, BFDS, and LBFDS appeared to yield significantly higher PRM (*P* < 0.001), the FUDS appeared to yield significantly lower PRM (*P* < 0.001), and no significant difference with LFDS was observed. Compared with the BRDS strategy, the FUDS, LBDS, LFDS, and LBFDS appeared to yield significantly lower PRM (*P* < 0.001), and no significant difference with BFDS was observed. Compared with the FUDS strategy, the LBDS, LFDS, BFDS, and LBFDS appeared to yield significantly higher PRM (*P* < 0.001). Compared with the LBDS strategy, the LFDS appeared to yield significantly lower PRM (*P* < 0.001), the BFDS appeared to yield significantly higher PRM (*P* = 0.003), and no significant difference with LBFDS was observed. Compared with the LFDS strategy, the BFDS and LBFDS appeared to yield significantly higher PRM (*P* < 0.001). When compared with the BFDS strategy, the LBFDS appeared to yield significantly lower PRM (*P* = 0.004).

**Table 4:** Range of motion in pelvic angle form dynamic change

		*P* value of the Wilcoxon signed-rank test					
DSSs	PRM (Degree)	LPDS	BRDS	FUDS	LBDS	LFDS	BFDS
LPDS	4.02 ± 1.84						
BRDS	39.70 ± 4.60	<0.001					
FUDS	0.93 ± 1.06	<0.001	<0.001				
LBDS	31.44 ± 5.10	<0.001	<0.001	<0.001			
LFDS	4.22 ± 2.26	0.823	<0.001	<0.001	<0.001		
BFDS	37.67 ± 8.68	<0.001	0.723	<0.001	0.003	<0.001	
LBFDS	31.31 ± 6.11	<0.001	<0.001	<0.001	0.852	<0.001	0.004
*P* value of the Friedman test				<0.001			

Comparison of mean pelvic range of motion (*PRM*) form dynamic change across 7 dynamic sitting strategies (*DSSs*), which include Lumbar Prominent Dynamic Sitting (*LPDS*), Back Reclined Dynamic Sitting (*BRDS*), Femur Upward Dynamic Sitting (*FUDS*), Lumbar Prominent with Back Reclined Dynamic Sitting (*LBDS*), Lumbar Prominent with Femur Upward Dynamic Sitting (*LFDS*), Back Reclined with Femur Upward Dynamic Sitting (*BFDS*), and Lumbar Prominent with Back Reclined with Femur Upward Dynamic Sitting (*LBFDS*). Each PRM parameter is given as the averaging value when two dynamic alteration process over a sitting trial. Values are mean ± standard deviation (*N* = 20)

## Discussion

Previous studies have proposed numerous dynamic devices for relieving pressure through periodically changing sitting positions; such devices include dynamic lumbar supports, dynamic reclined backrests, and dynamic ischial/femur cushions [20, 21, 24–27]. However, no study to date has examined whether combining these pressure-relieving devices produces a positive or negative effect. In the present study, we combined the aforementioned devices into 7 DSSs (ie, LPDS, BRDS, FUDS, LBDS, LFDS, BFDS, and LBFDS) and quantified their effects on the lumbar-pelvic angle to identify the effects and differences among these DSSs.

Lumbar curvature affects stress load on tissues including the ligaments, fascia, tendons, muscles, intervertebral discs, and vertebrae [1, 4, 31]. Both lumbar kyphosis and lordosis can result in uneven loading distribution on the anterior annulus fibrosus, nucleus pulposus, and posterior annulus fibrosus, and prolonged static load can cause metabolite accumulations of the intervertebral discs [1, 4, 22]. Many current studies propose periodic stimulation of lumbar movement to improve stress distribution in the intervertebral discs and metabolic transport as a means of preventing lower back pain [1, 20, 21, 27]. In addition, previous studies have shown that stress on the intervertebral discs increases with increased lumbar kyphosis but decreases with increased lumbar lordosis [1, 4, 32]. The sitting position of most wheelchair users naturally produces lumbar kyphosis [3, 10]. Thus, we believe that the most beneficial dynamic changes to sitting posture result in lumbar lordosis because increased lumbar lordosis decreases stress on the intervertebral discs. In this study, we measured LA and LRM to understand how different DSSs affect lumbar curvature and the direction and magnitude of these changes. Compared with the other DSSs, FUDS produced a significantly negative LA (ie, lumbar kyphosis) and the smallest LRM. Throughout all dynamic changes, FUDS resulted in only a small magnitude of lumbar kyphosis and may lead to the negative effects that accompany lumbar kyphosis. All other DSSs (ie, LPDS, BRDS, LBDS, LFDS, BFDS, and LBFDS) produced positive LA. The LBDS and LBFDS strategies, which are a combination of lumbar-prominent and back-reclined DSSs, produced significantly larger LRMs compared with the other DSSs, although no significant difference in LRM was found between these 2 DSSs. The next largest LRMs were produced by the LPDS and LFDS strategies, which are lumbar-prominent DSSs, and no significant difference in LRM was found between these 2 strategies. The third largest LRMs were produced by BRDS and BFDS, which are back-reclined DSSs, although no significant difference in LRM was observed between these 2 strategies. These findings imply that the ability of femur-upward DSSs to stimulate ideal lumbar movement is limited. However, previous studies have reported a positive correlation between ischial tuberosity pressure and spinal loading [2, 33, 34]. We believe that femur-upward DSSs can reduce peak pressure on the ischial tuberosity. However, interface pressure was not measured in the present study and this assumption requires further examination.

Because of the lumbar-pelvic rhythm, lumbar kyphosis occurs concurrently with posterior pelvic tilt and lumbar lordosis with anterior pelvic tilt [35]. Posterior pelvic tilt decreases the tightness of the hip extensors, but prolonged posterior pelvic tilt can cause irreversible shortening of the hip extensors and flexors [3, 36]. This then affects the range of motion of the lumbar and pelvis [3, 36]. Anterior pelvic tilt increases the hip extension tightness, but this lengthening of the muscles causes tension in the muscle [3, 36–38]. This tension increases the passive tensile loading on surrounding viscoelastic tissues, and prolonged tension can increase the risk of pain or discomfort [3, 4, 36]. Previous studies have suggested that periodic rotational movement of the pelvis can help activate muscle movement, and a greater range of motion leads to greater mitigation of the aforementioned negative effects [36]. In this study, we measured PA and PRM to understand how different DSSs affect pelvic tilt angle and the direction and magnitude of these changes. The results show that BRDS, FUDS, LBDS, BFDS, and LBFDS produced a significantly negative PA (ie, posterior pelvic tilt). The BRDS and BFDS strategies, both of which are back-reclined DSSs, produced the largest PRMs, and no significant difference in PRM was observed between these 2. The next largest PRMs were produced by LBDS and LBFDS, which are a combination of lumbar-prominent and back-reclined DSSs, and no significant difference in PRM was observed between these 2. The smallest PRM was produced by the FUDS strategy, which is a femur-upward DSS. As mentioned previously, lumbar-prominent and back-reclined DSSs result in greater lumbar lordosis, and because of the lumbar-pelvic rhythm, lumbar lordosis occurs concurrently with anterior pelvic tilt. Thus, a combination of lumbar-prominent and back-reclined DSSs produced a smaller PRM than back-reclined only DSSs did. LPDS and LFDS produced a positive PA (ie, anterior pelvic tilt) and smaller PRMs, and no significant difference was observed between these 2 strategies. Throughout all dynamic changes, LPDS and LFDS did not result in posterior pelvic tilt but resulted in a small magnitude of anterior pelvic tilt. These findings imply that the ability of LPDS and LFDS to stimulate ideal pelvic movement is limited.

Results show that among all the DSSs, LBDS and LBFDS resulted in the most beneficial lumbar movements, and no significant differences were observed between these 2 strategies. BRDS and BFDS resulted in the most beneficial pelvic movements, and no significant differences were observed between these 2 strategies. However, back-reclined DSSs, such as the BRDS, LBDS, BFDS, and LBFDS strategies, may affect normal daily functions and movements such as field of vision, eating, reaching for objects, or moving the wheelchair. Thus, back-reclined wheelchairs are mostly used clinically by patients with cerebrovascular accidents or frail older people. Wheelchair users who need to sit upright should choose LPDS or LFDS; among all the upright DSSs, they produced the most beneficial lumbar and pelvic movements, and no significant differences were observed between these 2 strategies.

A limitation of this study is that we recruited able-bodied participants instead of wheelchair users. We recruited these participants because prolonged testing and multiple chair transfers may present a physical burden and possible danger to wheelchair users. In future studies, we will reduce the testing time and select more meaningful DSSs that are practical and applicable to wheelchair users. In addition, Each DSS test lasted 20 min, but most of wheelchair users will stay in their chairs for prolonged time (more than 20 min). We assumed nonsignificant differences in lumbar and pelvic movements induced between DSS tests lasting 20 min and those lasting more than 20 min, although further research is needed in this regard.

## Conclusions

We identified the effects and differences among 7 DSSs on lumbar-pelvic angle. Wheelchair users can choose the most suitable DSS that meets their needs. These findings may serve as a reference for practicing physicians or wheelchair users to choose a dynamic wheelchair seating system. However, the present study examined only the overall angle in the lumbar and pelvis. Future studies that can determine the mechanics and physiological effects of dynamic changes on individual lumbar vertebra can provide a deeper understanding of the potential benefits of the different DSSs.

## Abbreviations

BFDS: Back reclined with femur upward dynamic sitting
BRDS: Back reclined dynamic sitting
DSSs: Dynamic sitting strategies
FUDS: Femur upward dynamic sitting
LA: Lumbar angle
LBDS: Lumbar prominent with back reclined dynamic sitting
LBFDS: Lumbar prominent with back reclined with femur upward dynamic sitting
LFDS: Lumbar prominent with femur upward dynamic sitting
LPDS: Lumbar prominent dynamic sitting
LRM: Lumbar range of motion
PA: Pelvic angle
PRM: Pelvic range of motion
